# The Power of Ground User in Recommender Systems

**DOI:** 10.1371/journal.pone.0070094

**Published:** 2013-08-02

**Authors:** Yanbo Zhou, Linyuan Lü, Weiping Liu, Jianlin Zhang

**Affiliations:** 1 Institute of Information Economy, Alibaba Business College, Hangzhou Normal University, Hangzhou, People’s Republic of China; 2 Department of Physics, University of Fribourg, Fribourg, Switzerland; Semmelweis University, Hungary

## Abstract

Accuracy and diversity are two important aspects to evaluate the performance of recommender systems. Two diffusion-based methods were proposed respectively inspired by the mass diffusion (MD) and heat conduction (HC) processes on networks. It has been pointed out that MD has high recommendation accuracy yet low diversity, while HC succeeds in seeking out novel or niche items but with relatively low accuracy. The accuracy-diversity dilemma is a long-term challenge in recommender systems. To solve this problem, we introduced a background temperature by adding a ground user who connects to all the items in the user-item bipartite network. Performing the HC algorithm on the network with ground user (GHC), it showed that the accuracy can be largely improved while keeping the diversity. Furthermore, we proposed a weighted form of the ground user (WGHC) by assigning some weights to the newly added links between the ground user and the items. By turning the weight as a free parameter, an optimal value subject to the highest accuracy is obtained. Experimental results on three benchmark data sets showed that the WGHC outperforms the state-of-the-art method MD for both accuracy and diversity.

## Introduction

The explosive growth of the Internet and WWW raises a serious information overload problem: we face too many data and resources to effectively find out the relevant ones by our limited processing abilities. How to measure the values of all the alternatives and then identify the useful information is a crucial problem, which asks for the development of advanced automatic techniques on information filtering. Search engines are useful tools, by which users can find the relevant information with properly chosen queries. However, they lack the consideration of personalization and thus return the same results to people no matter what their preferences are. Besides, since the search engines require the keywords extracted by the users themselves, when the users don’t know what they want or their preferences can’t be expressed by keywords, the search engines are of no avail. To address these problems, recommender systems rise in response to the proper time and conditions, which do not require specified keywords, instead they use the users’ historical activities and possible personal profiles to uncover their preferences and recommend the relevant items to the users according to their potential interests [1]. Actually, the recommendation can be considered as a link prediction problem on web-based user-item bipartite networks [2].

Many recommendation algorithms have been developed, including collaborative filtering [3], [4], content-based analysis [5], spectral analysis [6], [7] and iterative self-consistent refinement [8], [9]. What most have in common is that they are based on similarity, either of users or items or both. Such approach is under high risk of providing poor coverage of the space of relevant items. As a result, with recommendations based on similarity rather than difference, more and more users will be exposed to a narrow band of popular items, and niches items will be hard to excavate. Although it seems more accurate to recommend popular items than niche ones, being accurate is not enough [10]. Diversity and novelty are also important criteria of algorithmic performance. The diversity-accuracy dilemma becomes one of the main challenges in recommender systems.

Recently, some physical dynamics, including mass diffusion [11] and heat conduction process [12] have been applied to design recommender systems. It was shown that MD has high accuracy yet low diversity, while HC has high diversity yet low accuracy. To solve the accuracy-diversity dilemma, a hybrid method that combining HC and MD was proposed [13]. Other methods include the biased HC which improves the accuracy of HC while keeping its diversity [14], and the biased MD methods which improve the diversity of MD algorithm while keeping its accuracy [15], [16]. Different from the previous studies that mainly focused on the modification of the algorithms, in this paper we will show that the ground user who is supposed to select all the items in the system can improve the recommendation accuracy of HC while keeping its diversity. The ground user can also benefit other systems and its weighted form can further improve the performance.

## Materials and Methods

### Diffusion-based Methods

A recommender system can be represented by a user-item bipartite network 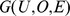, which consists of a set of users 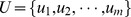, a set of items 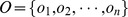, and a set of links between them 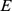. Denoted by 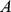 the adjacency matrix, where the element 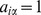 if user 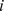 has collected the item 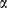, 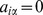 otherwise. We use Latin letters for users and Greek letters for items. The degree of item 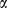 (i.e., the number of users who have collected the item 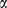) is denoted as 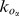 and the degree of user 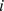 (i.e., the number of items that connect to user 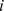) is denoted as 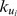. The essential task of a recommender system is to generate a ranking list of the target user’s uncollected items, based on the observed information.

The original diffusion-based methods is called mass diffusion (MD) which is based on the resource allocation process on the user-item bipartite network [11]. For a target user 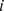, a certain amount of resource is assigned to each item that the user 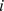 has collected. Since the network is unweighted (The biased allocation process was discussed in Ref. [15]), the unbiased allocation of the initial resource is split equally among all its neighboring users. Consequently, resources collected by users are equally redistributed back to their neighboring items. Denote by 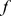 the initial resource vector where 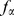 is the resource proposed by item 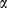. Recommendations for the target user 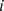 are obtained by setting 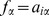. The redistribution is via transformation 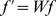, where
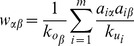
(1)is the resource transfer matrix. Physically, the diffusion is equivalent to a three-step random walk starting with 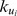 units of resource on the target user 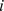. The recommendation score of an item is taken to be its amount of gathered resources after the diffusion. The resulting recommendation list of uncollected items is then sorted according to 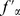 in descending order.

Different from MD, HC (we abbreviate this algorithm as HC, since it follows a conductive process analogous to heat diffusion across the user-item bipartite network) recommends items to an individual user by a process motivated by heat diffusion: items liked and disliked by this user are represented as hot and cold spots respectively, and recommendation is made according to the equilibrium temperature of the nodes in the networks [12]. The transition matrix of HC is represented by
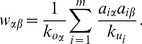
(2)


**Figure 1:**
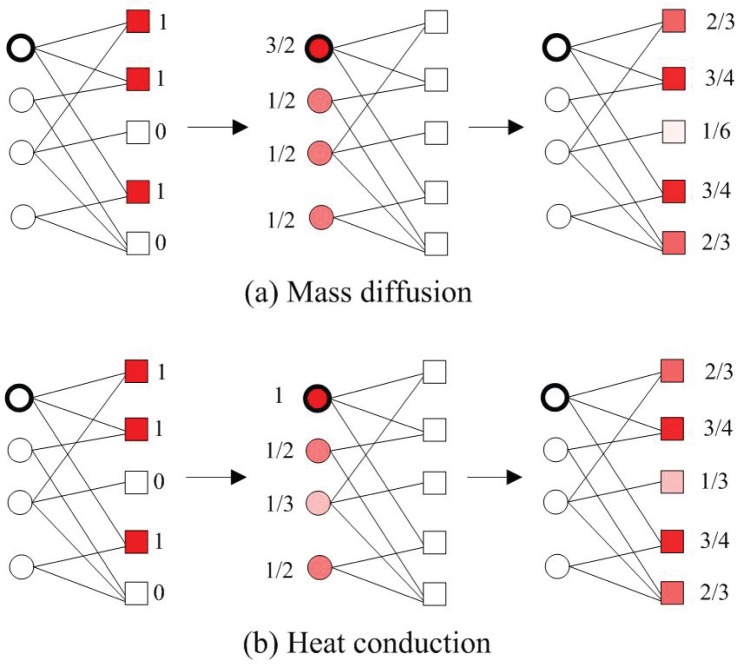
Illustrations of (a) MD and (b) HC algorithms. Users and items are presented by circles and squares respectively. The target user is labeled by thick edge. The color of a node indicates its amount of gathered resources at each step. The deeper the color is, the more resources it owns.

Similar to MD, HC also redistributes resources in a manner akin to a random-walk process. However the difference is significant in the diffusion process: the HC algorithm redistributes a resource via a nearest-neighbor averaging process, while the MD algorithm works by equally distributing the resource to the nearest neighbors. An illustration of the MD and HC processes is shown in fig. 1.

It has been pointed out that MD has high recommendation accuracy yet low diversity, while HC succeeds in seeking out novel or niche items and thus enhances the personalization of individual user recommendations but with relatively low accuracy. An effective way to solve the accuracy-diversity dilemma is to combine HC and MD by incorporating the hybridization parameter 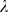 into the transition matrix normalization [13]:
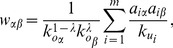
(3)where 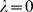 gives the pure HC algorithm and 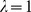 gives the MD algorithm. Such hybrid approach was shown to achieve both accurate and diverse recommendation subject to the optimal parameter 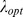.

**Figure 2:**
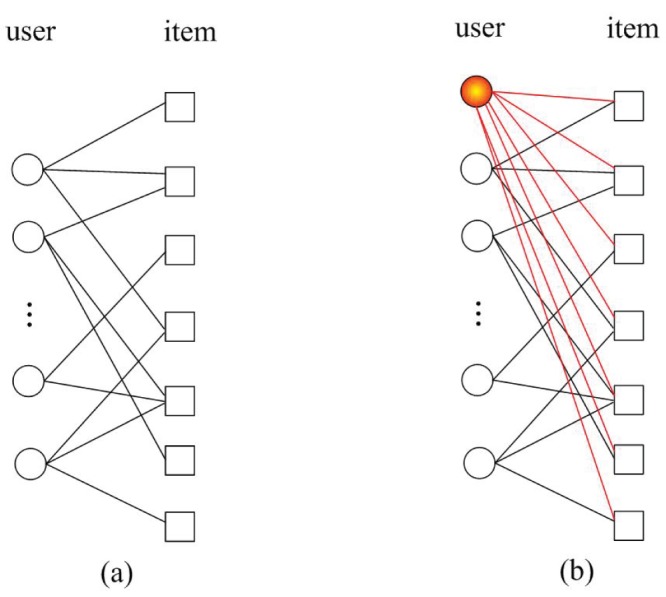
How to add a ground user to user-item bipartite network. Graph (a) is the original network. Graph (b) is the network after adding the ground user presented by filled circle. The red lines are the new added links between ground user and all the items.

Notice that the low-degree nodes are preferred in HC process than in MD process. For example in fig. 1, with MD the second user and the third user obtain the same recourse after one-step diffusion from the item side to the user side, while with HC the second user who owns lower degree 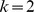 obtains more than the third user with 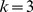. As a result, for the target user, the third item which is unpopular obtains more by HC. This is the reason why HC provides high diverse recommendation. A natural question is whether we can improve the recommendation accuracy while keep the diversity of HC. A potential way is the weighted HC where a turnable parameter is introduced [14]. Different from this route, we here propose a totally novel perspective where the key point is adding a ground user who collects all the items in the network. Figure 2 gives an example. The HC process will run on fig. 2(b) which consists of 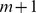 users and 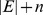 links. The transition matrix of the GHC (abbreviation of the HC with ground user) algorithm is thus written as
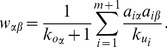
(4)


It can be rewritten as
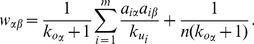
(5)


The first term is the contribution by the common users of items 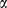 and 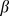, which is similar to the HC algorithm (see Eq. 2). The essential difference between HC and GHC lies in the second term of Eq. 5, which leads to an additional relation between two items even when they don’t have common users. It has been shown that the ground user can improve the performance on identifying influential leaders in social networks [17]. Here we will show that it also benefits the recommender systems. Experimental results show that by adding the ground user the recommendation accuracy will be largely increased. Clearly, for HC, the ground user only takes effect at the final step of the conductive process.

By assigning weight to each newly added link between the ground user and the item, we obtain a weighted form of HC algorithm with ground user (we abbreviate it as WGHC). In WGHC, the link between user 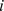 and item 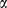 in the original data has the weight 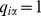, and the link between the ground user 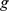 and item 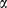 has the weight 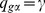. Thus, the transition matrix of WGHC is
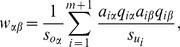
(6)where 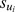 and 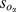 denote the weighted degree of user 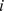 and item 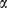, respectively. Equation 6 gives a weighted heat conduction process on a bipartite network. Clearly, when 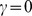, WGHC degenerates to HC, and when 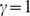, WGHC equals to GHC where the original links and the newly added links have the same weight. By tuning the parameter 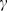, an optimal value 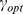 will be obtained subject to the highest accuracy.

### Methods for Comparison

For comparison, we present the results of three classical recommendation algorithms: the user-based K-Nearest-Neighbor (uKNN), item-based K-Nearest-Neighbor (iKNN), and weighted regularized matrix factorization (WRMF). KNN methods are very popular techniques in collaborative filtering. They rely on a similarity measure between either items (item-based) or users (user-based). In the uKNN method, for any user-item pair 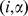, if user 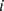 has not yet collected item 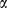, the predicted score 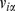 is given as
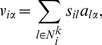
(7)where 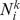 is the set of user 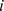
’s top-*k* nearest neighbors, and 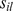 is the similarity between user 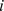 and user 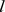. The main idea embedded in uKNN is that the target user will be recommended the items collected by those users sharing similar tastes with him. Different from uKNN, iKNN will recommend items similar to the ones that the target user preferred in the past. In iKNN method, the predicted score 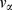 for user 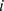 to item 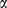 is defined as
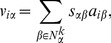
(8)where 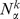 is the set of item 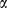
’s top-k nearest neighbors, and 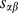 is the similarity between item 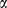 and item 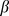. Here, we use cosine similarity to measure the similarity between users or items. Notice that if we use all their neighbors to calculate the predicted scores, that is 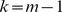 for uKNN and 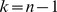 for iKNN, then uKNN and iKNN become respectively the standard user-based and item-based collaborative filtering algorithms, which will also be investigated in our experiments.

Weighted regularized matrix factorization [18], [19] is a matrix factorization method for item prediction. This method is an adaption of SVD. It associates each user 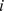 with a user-factors vector 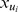, and each item 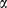 with an item-factors vector 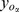. The prediction is done by taking an inner product of these two vectors, namely 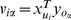. The factors are computed by minimizing the following cost function (i.e., the prediction error):
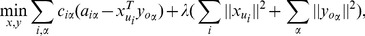
(9)where 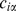 measures the confidence in observing 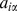. Zero value of 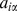 should be associated with low confidence, as not taking any positive action doesn’t mean that the user doesn’t like the item. The 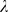 term is necessary for regularizing the model such that it will not overfit the training data. Here we set 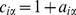 and 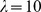.

### Data Description

We use three benchmark data sets, MovieLens, Netflix and RYM, to test the algorithmic performance. The MovieLens data set is provided by GroupLens project at University of Minnesota (www.grouplens.org). Here, we use the data with 1 million ratings by 6040 users on 3952 items. The ratings are given on the integer scale from 1 to 5 (i.e., worst to best). We here only consider the ratings higher than 2. That is if a user 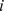 rates the item 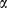 higher than 2, it means the user likes the movie and there will be a link between user 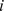 and item 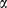 in the user-item bipartite network. After coarse gaining, the data contains 836478 links (i.e., user-item pairs). The Netflix data set is a huge data set released by the DVD rental company Netflix for its Netflix Prize (www.netflixprize.com). The ratings in Netflix are also given on the integer scale from 1 to 5. Similar to MovieLens data, only the links with ratings no less than 3 are considered. We extract a smaller data set by randomly sampling of the whole records of user activities. It finally consists of 10000 users, 6000 movies, and 701947 links. The RYM data set is publicly available on the music ratings website *RateYourMusic.com*. The ratings in RYM are given on the integer scale from 1 to 10. We here only consider the ratings higher than 5. The final data consists of 33221 users, 5234 albums, and 610398 links. Comparing with MovieLens and Netflix data sets, RYM is much sparser. Table 1 shows the basic statistical features of these three data sets.

**Table 1:** Basic statistical features of the data sets.

Data set	users	items	links	Sparsity
Movielens	6040	3952	836478	3.5×10^–2^
Netflix	10000	6000	701947	1.17×10^–2^
RYM	33221	5234	610398	3.50×10^–3^

### Evaluation Metrics

To test the algorithmic performance, the data is randomly divided into two parts: the training set 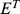 contains 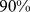 of the data and the remaining 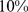 of the data constitutes the probe set 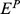. The recommendation list for each user is provided based on the training set, and the probe set will be used for testing. We apply six metrics to give quantitative measurements of the methods: ranking score, precision, normalized discounted cumulative gain (NDCG), intra-similarity, hamming distance and novelty.

Ranking score [11] is a metric for accuracy. It measures the ability of a recommendation algorithm to rank users’ preferable items higher places than the disliked ones. For a target user 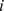, the recommender system will return a ranking list of all his uncollected items to him. Ranking score measures the relevant rank of each hidden items (i.e., items in probe set for user 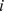) in the recommendation list of this user. For example, a hidden item 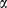 (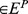) with ranking 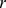 has the ranking score 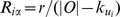, where 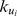 is the degree of user 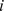 in 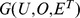. Averaging over all the hidden user-item relations, we obtain the mean value of ranking score, namely
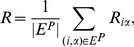
(10)where 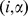 denotes the probe link connecting user 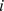 and item 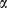. Clearly, the smaller the ranking score, the higher the algorithm’s accuracy.

Since in many real online systems, only the top part of the recommendation list is presented to users, therefore a more practical approach is to consider the number of a user’s relevant items ranked in the top-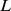 places. Precision is one of the popular measurements based on this. For a target user 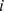, the precision of the recommendation is defined as
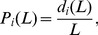
(11)where 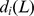 indicates the number of user 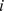
’s hidden items in the top-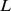 places of his recommendation list. The precision of the whole system 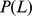 can be obtained by averaging the individual precisions over all users who have at least one hidden link. In this Letter, we set 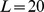.

Another measurement for rank capabilities in recommender systems is normalized discounted cumulative gain (NDCG) which has different discount gain in averaging the ranked items [20]. For a ranking list of a target user 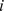
’s all uncollected items, the discounted cumulative gain (DCG) is defined as
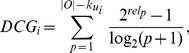
(12)where 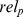 is the graded relevance of the result at position 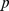. Here in our experiments, 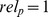 if the item at position 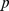 is the user’s hidden item, and 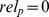 otherwise. Under this definition, we can see that DCG actually gives the hidden item at position 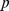 a score 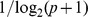. This is very similar to ranking score which assigns the hidden item at position 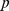 a score 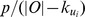. Therefore, if we divide DCG by the number of hidden items of a user, the obtained value will be negatively correlated with this user’s ranking score. Since ranking lists vary in length for different users, the DCG is normalized as
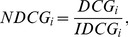
(13)where 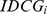 is the ideal DCG of the ranking list, which is the maximum possible DCG. Clearly, the higher the NDCG is, the better the ranking result is. The NDCG of the whole system can be obtained by averaging the individual NDCG over all users who have at least one hidden link.

Diversity is considered as another significant aspect for the evaluation of recommender systems. Hamming distance [21] is applied to measure the uniqueness of different users’ recommendation lists. Denoting 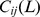 as the number of common items in the top-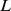 places of the recommendation lists of user 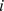 and 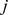, their hamming distance can be calculated as
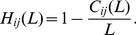
(14)


Clearly, 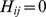 corresponds to the case where the recommendation lists of user 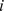 and user 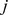 are exactly the same, while 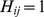 corresponds to the case where their lists are completely different. Averaging 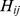 over all pairs of users, we obtain the mean distance 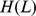. The greater the value is, the more diverse (or personalized) recommendations are given to the users.

Hamming distance only takes into account the diversity between users. However, a good algorithm is also expected to give diverse recommendation to a single user. Users may get tired of receiving many recommended items under the same topic [22]. Intra-similarity is proposed to measure the diversity of a user’s recommendation list [23]. For an arbitrary target user 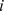 with a recommendation list 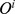, the intra-similarity is defined as
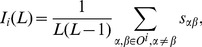
(15)where 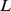 is the length of the recommendation list and 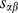 is the similarity of item 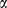 and item 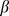. In this paper, we adopt the cosine similarity which is one of the most widely used similarity measures. For two items 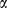 and 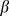, their cosine similarity is defined as



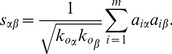
(16)The lower 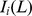 is, the more diverse items are recommended to the user. Averaging 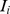 over all users, we obtain the mean intra-similarity 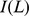 of the whole system.

Different from diversity which refers to how different the recommended items are with respect to each other, novelty measures the ability of an algorithm to generate unexpected and surprising recommendations. A good recommender system is expected to find the niche or unpopular items that cannot be easily known by other ways yet match users’ preferences. The simplest way to calculate novelty is to use the average popularity of the recommended items. Given a recommendation list 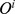 to user 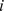 where 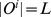, the novelty is defined as
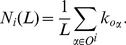
(17)


Lower 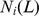 indicates higher novelty and surprisal. Averaging 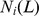 over all users, we obtain the mean novelty 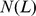 of the system.

## Results

The recommendation performances of different methods on the Movielens, Netflix and RYM data sets are shown in table 2, table 3 and table 4, respectively. All the data points are averaged over ten independent runs with different data divisions. GHC is an abbreviation of the method HC with a ground user. HHM refers to the hybrid method that combines HC and MD algorithms, namely Eq. 3. GHHM is an abbreviation of hybrid method with ground user. WGHC is the weighed version of GHC. uKNN and iKNN are respectively the user-based k-nearest-neighbor and item-based k-nearest-neighbor algorithms. uKNN(all) and iKNN(all) are the cases that consider all the neighbors, namely the standard user-based and item-based collaborative filtering algorithms, respectively. WRMF is the abbreviation of weighted regularized matrix factorization. For these parameter-dependent algorithms, the optimal parameter for each algorithm is set as the one corresponding to the lowest ranking score.

**Table 2:** The performance of different methods on Movielens data set.

method	*R*	*P*(20)	NDCG	*I*(20)	*H*(20)	*N*(20)
MD	0.1045	0.0977	0.4052	0.4644	0.5584	1976.6
HC	0.1085	0.0119	0.3077	0.0123	0.8273	43.1
GHC	0.0917	0.0773	0.3565	0.1295	0.9621	388.1
WGHC	0.0763	0.1256	0.4346	0.2847	0.9580	770.5
HHM	0.0748	0.1334	0.4234	0.2571	0.9329	953.2
GHHM	0.0739	0.1390	0.4496	0.3054	0.9361	1018.8
uKNN	0.0842	0.1451	0.4767	0.3979	0.8694	1451.2
iKNN	0.0758	0.1452	0.4642	0.3747	0.9228	1212.9
uKNN(all)	0.1159	0.0907	0.3887	0.4757	0.5242	2002.5
iKNN(all)	0.1019	0.1172	0.4293	0.4514	0.7409	1661.9
WRMF	0.0795	0.1417	0.4570	0.3905	0.8822	1400.4

The parameters are set as: 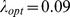 for HHM; 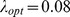 for GHHM; 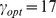 for WGHC; 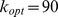 for uKNN; 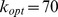 for iKNN.

**Table 3:** The performance of different methods on Netflix data set.

method	*R*	*P*(20)	NDCG	*I*(20)	*H*(20)	*N*(20)
MD	0.0502	0.0814	0.3846	0.4219	0.5546	2831.1
HC	0.1059	0.0002	0.2130	0.00538	0.8243	1.5
GHC	0.0609	0.0472	0.2829	0.0544	0.9103	549.3
WGHC	0.0460	0.0894	0.3956	0.2883	0.8230	1699.3
HHM	0.0449	0.0952	0.4022	0.3313	0.7578	2209.8
GHHM	0.0442	0.0976	0.4080	0.3322	0.7713	2140.9
uKNN	0.0502	0.1004	0.4213	0.4064	0.7107	2562.7
iKNN	0.0484	0.0993	0.4084	0.4029	0.7466	2363.1
uKNN(all)	0.0584	0.0767	0.3732	0.4326	0.5142	2874.9
iKNN(all)	0.0524	0.0858	0.3821	0.4284	0.6420	2446.1
WRMF	0.0448	0.0984	0.3883	0.3290	0.7468	1957.9

The parameters are set as: 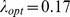 for HHM; 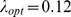 for GHHM; 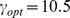 for WGHC; 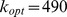 for uKNN; 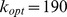 for iKNN.

**Table 4:** The performance of different methods on RYM data set.

method	*R*	*P*(20)	NDCG	*I*(20)	*H*(20)	*N*(20)
MD	0.0613	0.0671	0.4176	0.1845	0.7296	1828.4
HC	0.0673	0.0430	0.3152	0.0658	0.9296	275.8
GHC	0.0611	0.0533	0.3651	0.1004	0.9511	360.4
WGHC	0.0509	0.0649	0.4267	0.1586	0.9428	548.1
HHM	0.0452	0.0731	0.4601	0.1588	0.9353	846.0
GHHM	0.0452	0.0731	0.4613	0.1615	0.9354	840.6
uKNN	0.0692	0.0612	0.4008	0.1953	0.7406	1788.2
iKNN	0.0536	0.0669	0.4556	0.2076	0.9534	782.6
uKNN(all)	0.0869	0.0540	0.3690	0.1999	0.6865	1910.0
iKNN(all)	0.0677	0.0630	0.4499	0.2149	0.9386	881.3
WRMF	0.0755	0.0482	0.3458	0.2038	0.8933	1162.5

The parameters are set as: 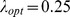 for HHM; 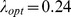 for GHHM; 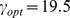 for WGHC; 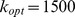 for uKNN; 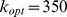 for iKNN.

**Figure 3:**
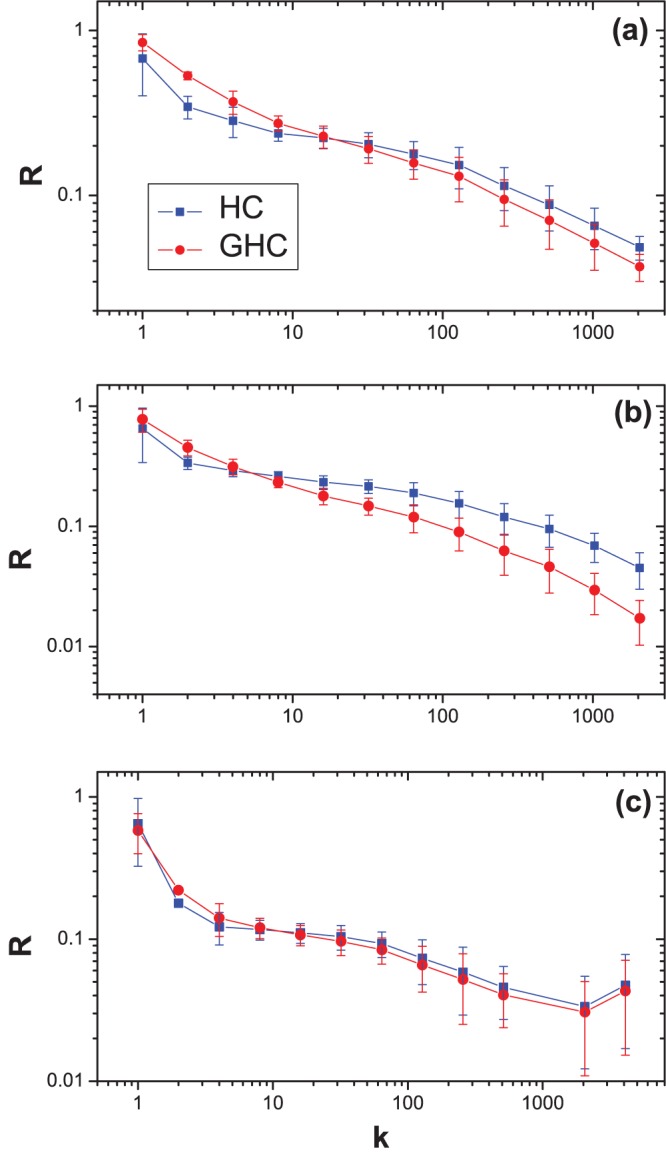
The dependence of ranking score 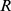 on the item degree 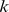. Graphs (a), (b) and (c) are respectively the results on Movielens data set, Netflix data set and RYM data set. The squares and circles present the HC and GHC algorithms, respectively.

Comparing the results of HC and GHC, we can see that the recommendation accuracy can be improved by adding a ground user for all three data sets. Especially, the improvement is significant when we focus on the precision of top-20 recommended items for Netflix and Movielens. The 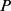
(20) increases from 0.0119 to 0.0773 for MovieLens data set, and increases from 0.0002 to 0.0472 for Netflix data set. The improvement mainly comes from the accurate recommendations on popular items. Figure 3 shows the dependence of ranking score on the item degree of HC and GHC algorithms. Previous studies have shown that the original HC algorithm prefers to the small-degree items (i.e., unpopular items), which is supported by the very small average degree of the recommended items, see 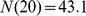 for Movielens, 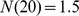 for Netflix, and 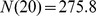 for RYM. While by adding a ground user, the bias can be relieved. The main contribution of the ground user is to add an additional transition probability from one item to another. Actually, the number of heat source of ground user can be considered as the temperature of the whole system. Each item receives the same heat from the ground user and then average it with the heat from other sources. As a result, the temperature of the popular items will be enhanced. Besides accuracy, the ground user also improves the inter-diversity of the recommendation results (see the improvement of hamming distance by GHC) while keeping a relatively high novelty.

**Figure 4:**
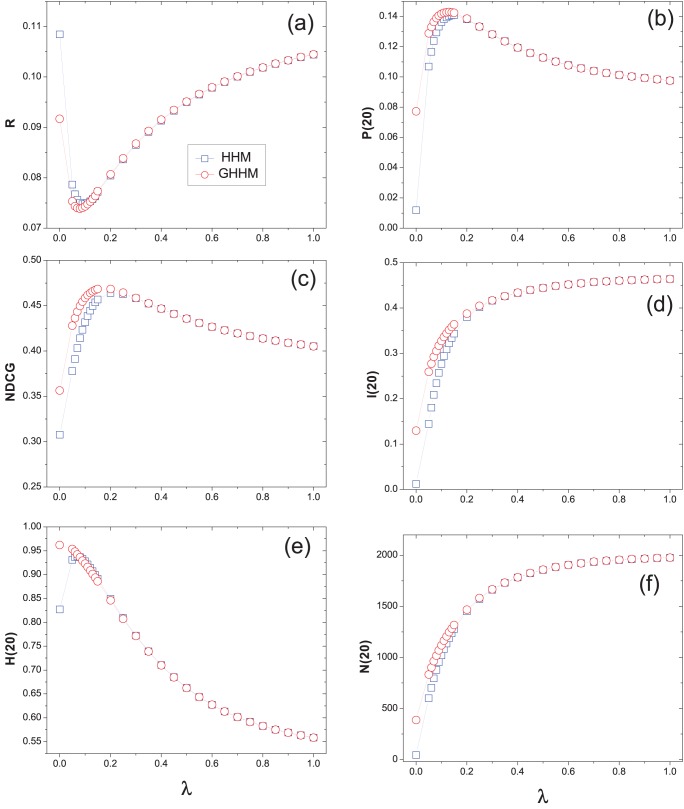
The recommendation results of hybrid method on Movielens data set. The original hybrid method (i.e., HHM ) is presented by squares and the hybrid method with a ground user (i.e., GHHM) is presented by circles.

**Figure 5:**
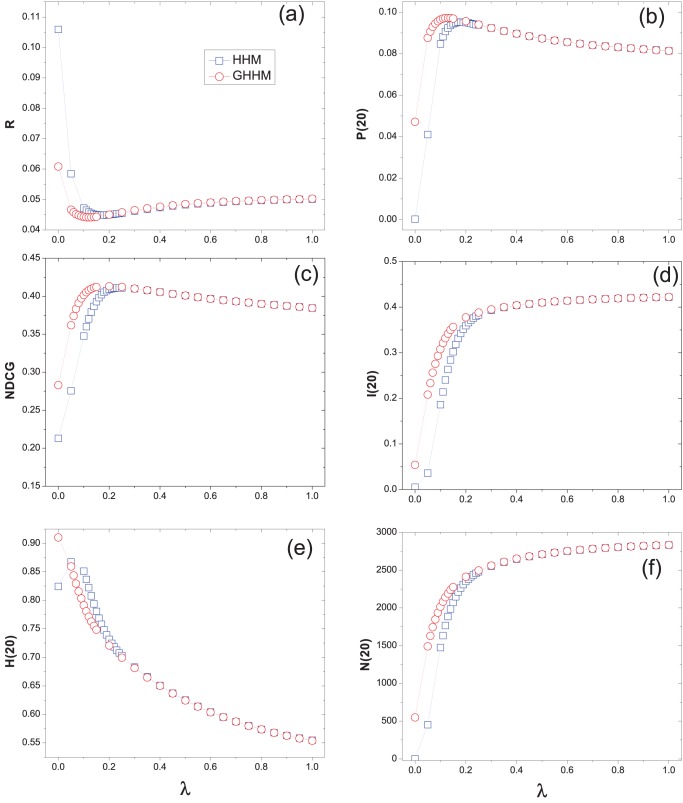
The recommendation results of hybrid method on Netflix data set. The original hybrid method (i.e., HHM ) is presented by squares and the hybrid method with a ground user (i.e., GHHM) is presented by circles.

**Figure 6:**
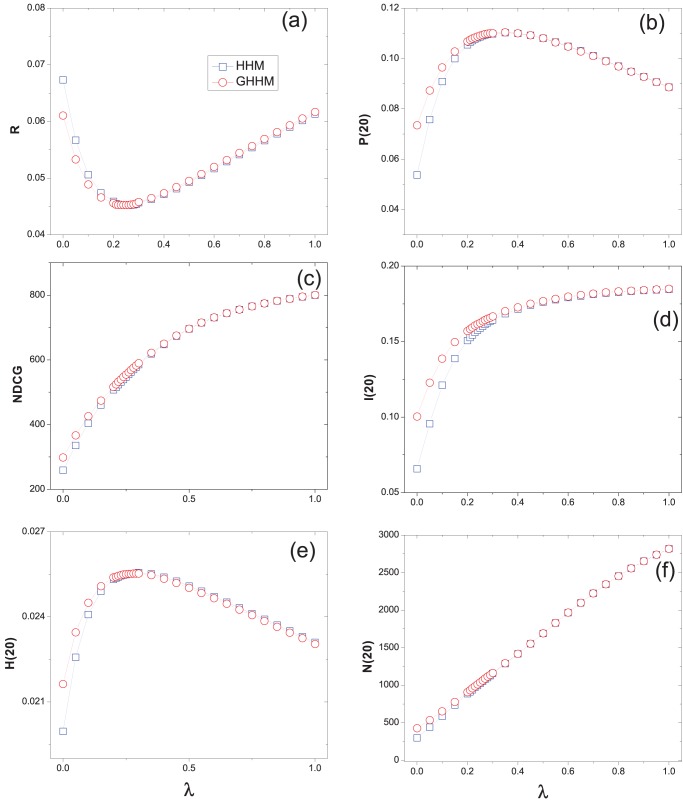
The recommendation results of hybrid method on RYM data set. The original hybrid method (i.e., HHM ) is presented by squares and the hybrid method with a ground user (i.e., GHHM) is presented by circles.

We have known that the original hybrid method HHM is a good trade-off of diversity and accuracy of recommendation [13]. From our experiments, we find that the ground user also improves the accuracy of HHM while keeping high diversity and novelty. Figures 4, 5 and 6 show the performance of the hybrid algorithm under different 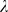 on Movielens, Netflix and RYM data sets, respectively. All the data points are averaged over ten independent runs with different data divisions. As we can see, for large 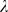 (indicates that the MD algorithm has a larger weight in the hybrid method) the HHM and GHHM perform almost the same. While given a small 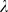 (indicates that the HC algorithm has a larger weight in the hybrid method) GHHM obtains lower ranking score than HHM. The optimal 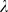 for the GHHM is smaller than that of the HHM method, meaning that the GHHM reaches the optimal case by considering less weight of MD and more weight of HC algorithm. That is also the reason why GHHM can slightly increase the recommendation diversity.

**Figure 7:**
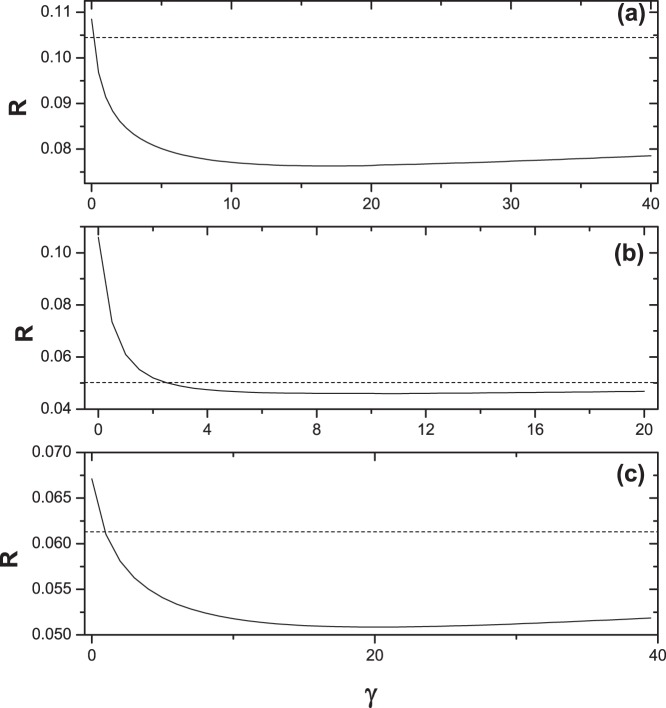
The ranking score 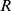 of WGHC algorithm with different weight parameter 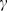. Graphs (a), (b) and (c) are the results on Movielens data set, Netflix data set and RYM data set, respectively. The dash lines are the ranking score of the MD method. All the data points are averaged over ten independent runs with different data divisions.

Now we consider the case when the weights of the newly added links between the ground user and items are different from the original ones, namely the weighted GHC method (WGHC), see Eq. 6. Figure 7 shows the dependence of ranking score of WGHC algorithm on parameter 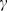. With the increasing of 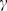, the ranking score of HC method decreases sharply at the beginning and then reaches the lowest point. As we discussed above, the ground user can reduce the preference of low-degree items of the HC method by adding additional relations to every pair of items. Assigning higher weight to the newly added links between the ground user and the items can enhance the influence of the additional relations. The WGHC method outperforms the MD algorithm for both accuracy and diversity. The improvements are significant. The exact scores of the six metrics on three data sets are given in tables 2, 3 and 4, respectively.

From the last five classical methods, it can be seen that, comparing with the standard user-based and item-based collaborative filtering algorithms, their corresponding KNN methods give better results in all six metrics. In general, iKNN performs better than uKNN. For MovieLens and RYM data sets, iKNN provides more accurate recommendations than WRMF in all three accuracy metrics, while in Netflix data set iKNN wins in precision and NDCG but WRMF has lower ranking score. Among all the eleven algorithms, GHHM yields the lowest ranking score on all three data sets. Besides, the diffusion-based methods are more efficient. In the worst case the complexity of one recommendation is approximate to 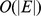, where 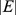 is the number of links [15].

Essentially, the diffusion-based methods (e.g., MD, HC and HHM) and the similarity-based methods (e.g., uKNN and iKNN) can be unified in a common framework, since they all work via a transformation 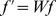. The main difference between these two groups of methods is how to define the matrix 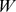. For uKNN and iKNN, 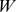 is actually the similarity matrix which is symmetric. While for diffusion-based methods, the transformation matrix 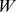 is asymmetric. The role of the ground user is to add an additional relation between two items. Whether the ground user can improve the performance depends on how it works on matrix 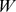. We have tested that the ground user will not affect the result of uKNN. The reason is twofold. On one hand, the cosine similarity between any two users will not change after adding the ground user. On the other hand, the ground user is hard to be included in the set of top-*k* nearest neighbors due to its very small similarity with the target user. For iKNN, the result becomes even worse with the consideration of a ground user. It is absurd that the similarity between two low-degree items which have not been selected by any common user changes from 0 to a high value after adding a ground user. This fact leads to a ridiculous result that the most dissimilar items are selected as top-*k* nearest neighbors.

## Discussion

To summary, we proposed a novel way to address the accuracy-diversity dilemma in recommender systems by adding a ground user who is supposed to select all the items and thus can be considered as the global environment or background temperature of the system. The main contribution of the ground user is to add an additional relation between every two items even when they don’t have any common users. Each item receives the same heat from the ground user and then average it with the heat from other sources. Comparing with the original heat conduction algorithm the temperature of the popular items will be enhanced. That is to say, the ground user can relieve the bias of the original heat conduction algorithm on unpopular items. Experiments on three benchmark data sets showed that the ground user can improve the accuracy while keeping high diversity, and especially the improvement is significant with its weighted form.

In the BIG DATA era, we are able to quantitatively characterize the Internet evolution and human online activities, which may result in large improvement of the technologies of information services and thus significant social and economic values. How to effectively find the relevant information within a huge data space is a crucial problem, with three key scientific issues: (i) Understanding the structure and evolution of information systems, as well as the originality and spreading dynamics of information. (ii) Understanding the spatio-temporal statistics of human online behaviors, as well as the correlation between users’ short-term and long-term interests embodied by their activities. (iii) Understanding the generation and organization of information, and providing better information services about prediction, navigation and recommendation. These studies promote the development of a new branch of research domains named “Infophysics”. In our future studies, we will keep working in this direction and apply the perspectives, theories and methods in statistical physics to develop efficient algorithms, uncover the statistical features hidden in the huge amount of data, summarize the universal law of the evolution of information systems and the behaviors of humans, and eventually provide advanced information services. We believe these studies will ultimately contribute to the science and engineering in the big data era.
